# Dynamic interplay between social distancing duration and intensity in reducing COVID-19 US hospitalizations: A “law of diminishing returns”

**DOI:** 10.1063/5.0013871

**Published:** 2020-07-15

**Authors:** Pai Liu, Payton Beeler, Rajan K. Chakrabarty

**Affiliations:** 1 Complex Aerosol Systems Research Laboratory, Department of Energy, Environmental and Chemical Engineering, Washington University in St. Louis, St. Louis, Missouri 63130, USA; 2 Institute for Public Health, Washington University in St. Louis, St. Louis, Missouri 63130, USA

## Abstract

We uncover and highlight the importance of social distancing duration and intensity in lowering hospitalization demand-to-supply during the coronavirus disease 2019 (COVID-19) epidemic in the USA. We have developed an epidemic progression model involving the susceptible–exposed–infected–recovered dynamics, the age-stratified disease transmissibility, and the possible large-scale undocumented (i.e., asymptomatic and/or untested) transmission of COVID-19 taking place in the USA. Our analysis utilizes COVID-19 observational data in the USA between March 19 and 28, corresponding to the early stage of the epidemic when the impacts of social distancing on disease progression were yet to manifest. Calibrating our model using epidemiological data from this time period enabled us to unbiasedly address the question “How long and with what intensity does the USA need to implement social distancing intervention during the COVID-19 pandemic?” For a short (i.e., up to two weeks) duration, we find a near-linear decrease in hospital beds demand with increasing intensity (φ) of social distancing. For a duration longer than two weeks, our findings highlight the diminishing marginal benefit of social distancing, characterized by a linear decrease in medical demands against an exponentially increasing social distancing duration. Long-term implementation of strict social distancing with φ>50% could lead to the emergence of a second wave of infections due to a large residual susceptible population which highlights the need for contact tracing and isolation before re-opening of the economy. Finally, we investigate the scenario of intermittent social distancing and find an optimal social-to-no-distancing duration ratio of 5:1 corresponding to a sustainable reduction in medical demands.

Social distancing has been adopted as a non-pharmaceutical intervention to prevent the coronavirus disease 2019 (COVID-19) pandemic from overwhelming the medical resources across the USA. The catastrophic socio-economic impacts of this intervention could outweigh its benefits if the timing and duration of implementation are left uncontrolled and ill-strategized. Here, we address these concerns by investigating the dynamics of three social distancing strategies—indefinite, finite-duration, and intermittent—on age-stratified US population and benchmark its effectiveness in reducing the burden on hospital beds. We find that a wholesale, indefinite social distancing could balance the demand-to-supply ratios of hospital beds at epidemic peaks provided a 70% reduction in the time-of-exposure of the population within all age groups is achieved. We find that finite-duration social distancing manifests itself into two distinct regimes. For social distancing lasting less than two weeks, a higher reduction in the time-of-exposure of the population results in less hospitalizations. For social distancing lasting longer than two weeks, ∼50% reduction in the time-of-exposure of the population yields the largest reduction in hospitalizations. Greater than this threshold reduction intensity, our analysis shows a large buildup of susceptible individuals, who remain prone to the infection at the end of the social distancing period. Overall, we emphasize the exponentially diminishing medical cost benefits of finite-duration social distancing—a phenomenon characterized by a linear decrease in medical demand achieved against an exponentially increasing social distancing duration. We finally investigate intermittent social distancing as a strategy to circumvent this situation and find a critical intermittent social-to-no-distancing duration ratio of 5:1, which facilitates up to 80% reduction in medical demands. Exceeding this critical ratio, the marginal benefit of social distancing wanes off significantly.

## I. INTRODUCTION

In December 2019, a novel coronavirus named SARS-CoV-2 began infecting residents of Wuhan, China.^1–3^ SARS-CoV-2 causes moderate to severe respiratory symptoms that can progress to severe pneumonia (coronavirus disease 2019, COVID-19).^4^ Despite the extreme disease containment measures taken in China,^5^ COVID-19 has spread rapidly to numerous countries and evolved into a global pandemic.^1,3^ On January 30, 2020, the World Health Organization (WHO) declared a “public health emergency of international concern,”^6^ and on the following day the United States Department of Health and Human Services declared a public health state of emergency.^7^ During the week of February 23, the US Centers for Disease Control (US-CDC) reported new confirmed cases of COVID-19 in California, Oregon, and Washington, indicating the onset of “community spread” across the USA.^7^ Until March 2, the total number of confirmed active COVID-19 cases in the USA were 33 with new cases emerging in the states of Texas, Arizona, Wisconsin, Illinois, Florida, New York, Rhode Island, and Massachusetts.^8^ In the following two weeks, this number rapidly increased from 527 confirmed cases on March 9 to 4216 cases on March 16.^8^ The states of California and New York declared a state emergency on March 4 and 7, respectively.^9,10^ The White House declared a national emergency on March 13^11^ foreseeing the inevitability of a major epidemic outbreak across the USA. As of May 14, the total number of confirmed cases in the USA has exceeded 1.4 × 10^6^.^8^ Based on past estimates of seasonality, immunity, and cross-immunity for betacoronaviruses OC43 and HKU1, it is highly likely that recurrent wintertime outbreaks of SARS-CoV-2 will occur after the initial, most serve pandemic wave in the USA.^12^

To prevent the rapid disease spread and alleviate the medical demands, social distancing has been adopted as a non-pharmaceutical measure across the country. Such a non-pharmaceutical intervention aims to slow down epidemic progression and ultimately prevents the country's medical system from collapsing due to overburdening of COVID-19 patients. It has also been suggested that prolonged or intermittent social distancing along with expanded critical care may be necessary until 2022.^12^ However, effectiveness and marginal benefits of social distancing as a function of implementation duration, timing, and strategy, especially in the context of COVID-19 epidemic, remain uncertain and is a critical knowledge gap.

Here, we address this gap by comprehensively investigating the effectiveness of social distancing as a function of implementation duration and intensity in reducing the peak number of hospitalizations^13^ across the USA. We do so by calibrating an epidemic dynamic model with COVID-19 observational data from the USA for the period March 19–28. This period was characterized by a rapid surge in COVID-19 cases due to a ramp up in testing services and the onset of strict social distancing in most US states. More importantly, the benefits of social distancing in reducing the hospital bed demand-to-supply were yet to manifest during this period, which facilitated us to unbiasedly address the research questions posed as part of this study.

## II. EPIDEMIC DYNAMIC MODEL AND CALIBRATION PERIOD

Our metapopulation epidemiological model involves the susceptible, exposed, infected, and recovered (SEIR) dynamics,^1,2,14–16^ the age-stratified disease transmissibility,^16–18^ and the possible large-scale undocumented transmission^16,19^ taking place in the 50 US states, Washington DC, and Puerto Rico (hereafter, they are generically denoted as states). For each state *n*, the local population was classified into four categories—susceptible, exposed, infected, and recovered—with the fraction of population within each category denoted as $s_{n},\;e_{n},\;j_{n}\;\text{and}\;r_{n}$, respectively.^20^ The infected category was further divided into two sub-classes: the infected-and-documented $\left(j_{n}^{\;r}\right)$ and the infected-and-undocumented $\left(j_{n}^{u}\right)$, i.e., $j_{n}=j_{n}^{\;r}+j_{n}^{u}$. This treatment accounts for the substantial influence of the asymptomatic (or mildly symptomatic) COVID-19 carriers on accelerating the epidemic spread.^19^ Furthermore, the population within each category of $s_{n},\;e_{n},\;j_{n}^{\;r},\;j_{n}^{u},\;\text{and}\;r_{n}$ were divided into nine age-stratified compartments (hereafter age groups), in light of the strong age-dependent hospitalization rate of COVID-19 patients.^13^ Specifically, age group *i* (with 1≤i≤8) comprised of individuals aged between 10 × (i−1) and 10 × *i *− 1 years, while the 9th age group included everyone aged 80 years and above. This age-stratification can be expressed as $x_{n}=\sum_{i}x_{n,i}$ (here, *x* is used to generically denote $s,\;e,\;j^{r},\;j^{u},\;\text{and}\;r$). Finally, the interstate exchange of individual within the $s_{n},\;e_{n},\;j_{n}^{u},\;\text{and}\;r_{n}$ category was captured using a mobility matrix of $P_{m,n}$ quantifying the probability that an individual leaving state *n* ends up in *m*.^20–22^ The governing equations of our model can therefore be expressed as a set of first-order differential equations with respect to time (t),

$$\frac{\partial }{\partial t}s_{n,i}=-\frac{R_{0,n,i}}{D_{I}}s_{n,i}j_{n}^{\;r}-\frac{R_{0,n,i}}{\varepsilon D_{I}}s_{n,i}j_{n}^{u}+\frac{f_{n,i}\phi }{\Omega }\sum_{m\neq n}P_{m,n}(s_{m,i}-s_{n,i}),$$

$$\begin{array}{rl} \frac{\partial }{\partial t}e_{n,i} & =\frac{R_{0,n,i}}{D_{I}}s_{n,i}j_{n}^{\;r}+\frac{R_{0,n,i}}{\varepsilon D_{I}}s_{n,i}j_{n}^{u}-\frac{1}{D_{E}}e_{n,i}\\ & \;+\frac{f_{n,i}\phi }{\Omega }\sum_{m\neq n}P_{m,n}(e_{m,i}-e_{n,i}), \end{array}$$

$$\frac{\partial }{\partial t}j_{n,i}^{r}=\frac{\eta_{n}}{D_{E}}e_{n,i}-\frac{1}{D_{I}}j_{n,i}^{r},$$

$$\frac{\partial }{\partial t}j_{n,i}^{u}=\frac{1-\eta_{n}}{D_{E}}e_{n,i}-\frac{1}{\varepsilon D_{I}}j_{n,i}^{u}+\frac{f_{n,i}\phi }{\Omega }\sum_{m\neq n}P_{m,n}(j_{m,i}^{u}-j_{n,i}^{u}),$$

$$r_{n,i}=f_{n,\;i}-e_{n,i}-j_{n,i}^{r}-j_{n,i}^{u}-s_{n,i}.$$ (1)

Here, $R_{0,n,i}$ is an age-specific reproduction ratio representing the efficiency by which COVID-19 (within state n) is transmitted to the local population in age group *i*. The values of $R_{0,n,i}$ were mapped from a state-wise reproduction ratio $\left(R_{0,n}\right)$ per the assumption that the disease transmissibility between age groups iandk is directly proportional to the average daily time-of-exposure $\left(T_{i,k}\right)$ among their members: $R_{0,n,i}=R_{0,n}\sum_{k}T_{i,k}/q$, where *q* is a proportionality factor taking values of the largest eigenvalue of the $T_{i,k}$ matrix.^17,18,23^ $D_{E}\;\text{and}\;D_{I}$, respectively, represent the mean incubation period and mean infectious period of COVID-19;^1,2,24^ $\eta_{n}$ represents local documentation ratio of the infected individuals; ε is a constant factor denoting the mean elongation of infectious period for those undocumented COVID-19 carriers;^19^ $f_{n,i}$ is the fraction of local population within age group i,ϕ is the daily passenger flux of the entire traffic network; and Ω represents the total US population.^20,25^ The ratio $f_{n,i}\phi /\Omega$ can be regarded as an interstate mobility parameter for members of age group *i*.

Our model was initialized on March 19 $\left(t_{0}\right)$ with the documented active COVID-19 cases $j_{n}^{\;r}(t_{0})$ acquired from a web-based dashboard for real-time epidemic tracking published by Johns Hopkins University.^8^ Next, we estimated the initial number of undocumented cases, per $j_{n}^{u}(t_{0})=(1/\eta_{n}-1)*j_{n}^{\;r}(t_{0})$, and the exposed cases, per $e_{n}(t_{0})=(1/\zeta_{n})*j_{n}^{\;r}(t_{0})\;$. (Here, $\zeta_{n}$ is the unknown documented-to-exposed ratio and $\eta_{n}$ is the unknown documentation ratio for state *n*. The acquisition of $\zeta_{n}\;\text{and}\;\eta_{n}$ is detailed below.) Finally, the age-stratified compartments were initialized according to the state-wise demographic composition, i.e., $x_{n,i}(t_{0})=f_{n,i}x_{n}(t_{0})$. The mobility matrices $P_{m,n}$ and passenger flux ϕ were calculated using the latest monthly resolved aviation data (between September 2018 and August 2019) released by the United States Bureau of Transportation Statistics^26^ (refer to supplementary material for details). It was assumed in our model that the interstate exchange of passengers is predominately via air traffic since volume of ground-based exchange is negligible (see the supplementary material). We further assumed that the long-term international importation of individuals infected with COVID-19 is minimal under a wholesale travel restriction enforced on international passengers that arrive from countries and regions where COVID-19 is widespread.^27–29^ The state-wise demographic composition $f_{n,i}$ was acquired from the database of the United States Census Bureau.^30^ The matrix of $T_{i,k}$ was acquired from the work by Zagheni *et al*.^17^ wherein the age-specific time-of-exposure was calculated using American Time Use Survey data (supplementary material).

The epidemiological parameters $D_{E},\;D_{I}\;\text{and}\;\varepsilon$ of COVID-19 were assumed to be 5.2 days, 2.3 days,^31^ and 1.82,^2^ respectively, per the values reported in the latest modeling study conducted on COVID-19 epidemics in China.^2,31^ For simplicity, we assumed here that the country-wise variability in the incubation and infectious periods of coronavirus is insignificant*.* The unknown state-wise parameters $R_{0,n},\;\eta_{n},\;\text{and}\;\zeta_{n}$ were inferred in a trial-and-error manner by fitting the model predicted $j_{n}^{\;r}(t)$ to the observational data within a calibration period. Specifically, the inference algorithm operates by iteratively guessing values of $R_{0,n},\;\eta_{n}\;\text{and}\;\zeta_{n}$ for each state *n*. The ranges for parameters—$R_{0,n}\in [0,10],\;\eta_{n}\in [0,0.4]\;\text{and}\;\zeta_{n}\in [0,1]$—were chosen based on values deemed realistic before widespread testing of COVID-19 was conducted.^2,19,32^ This iteration process is repeated until attaining the optimal $R_{0},\;\eta ,\;\text{and}\;\;\zeta$ arrays that minimize the root-mean-squared-error (RMSE) between the model predicted $j_{n}^{\;r}(t)$ and the ground truth.

**FIG. 1.**
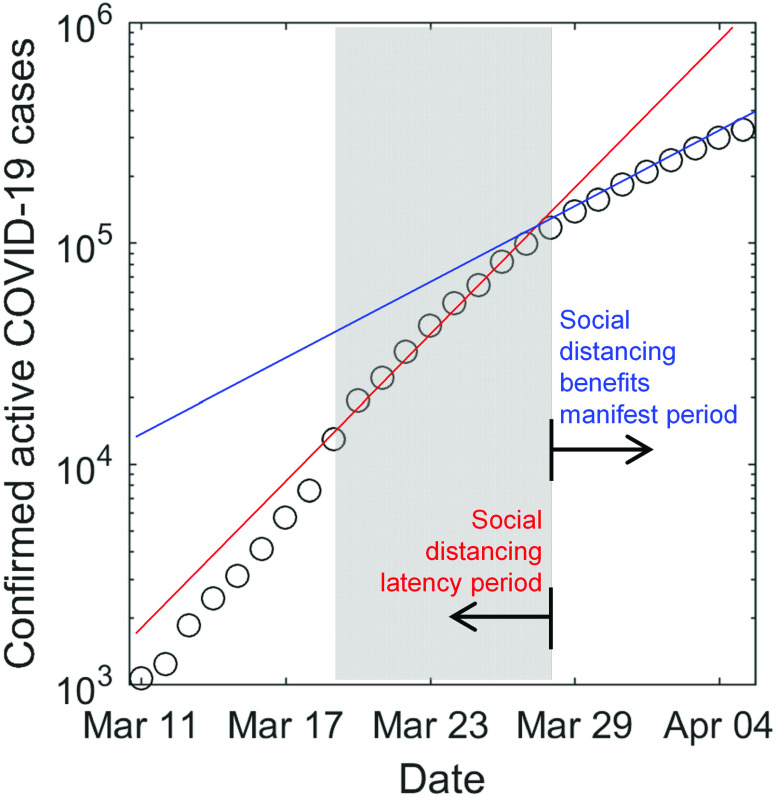
Evolution of epidemic size in the USA between March 10 and April 7. A distinct inflection point can be observed on March 28, characterized with a transition from a *social distancing latency period* regime to a *social distancing benefits manifest* regime.

The calibration period was set to the ten-day period between March 19 and 28, a period characterized by a rapid surge in epidemic size with a basic reproduction ratio $R_{0}\approx 4.87$ (state-wise median value) and the onset of social distancing in most US states in conjunction with ramp up in testing services. However, the benefits of social distancing in reducing the hospital bed demand-to-supply only started to manifest after March 28. Therefore, we term the period March 19–28 as the “latency period” (see the shaded area in Fig. 1), and the period thereafter as “benefits manifestation period.” Calibrating our model using baseline epidemiological data corresponding to the “latency period” enabled us to unbiasedly probe and address the question: *How long and with what intensity does the US need to implement social distancing as a sustainable public policy during the COVID-19 pandemic*?

## III. RESULTS

### A. Model calibration

**FIG. 2.**
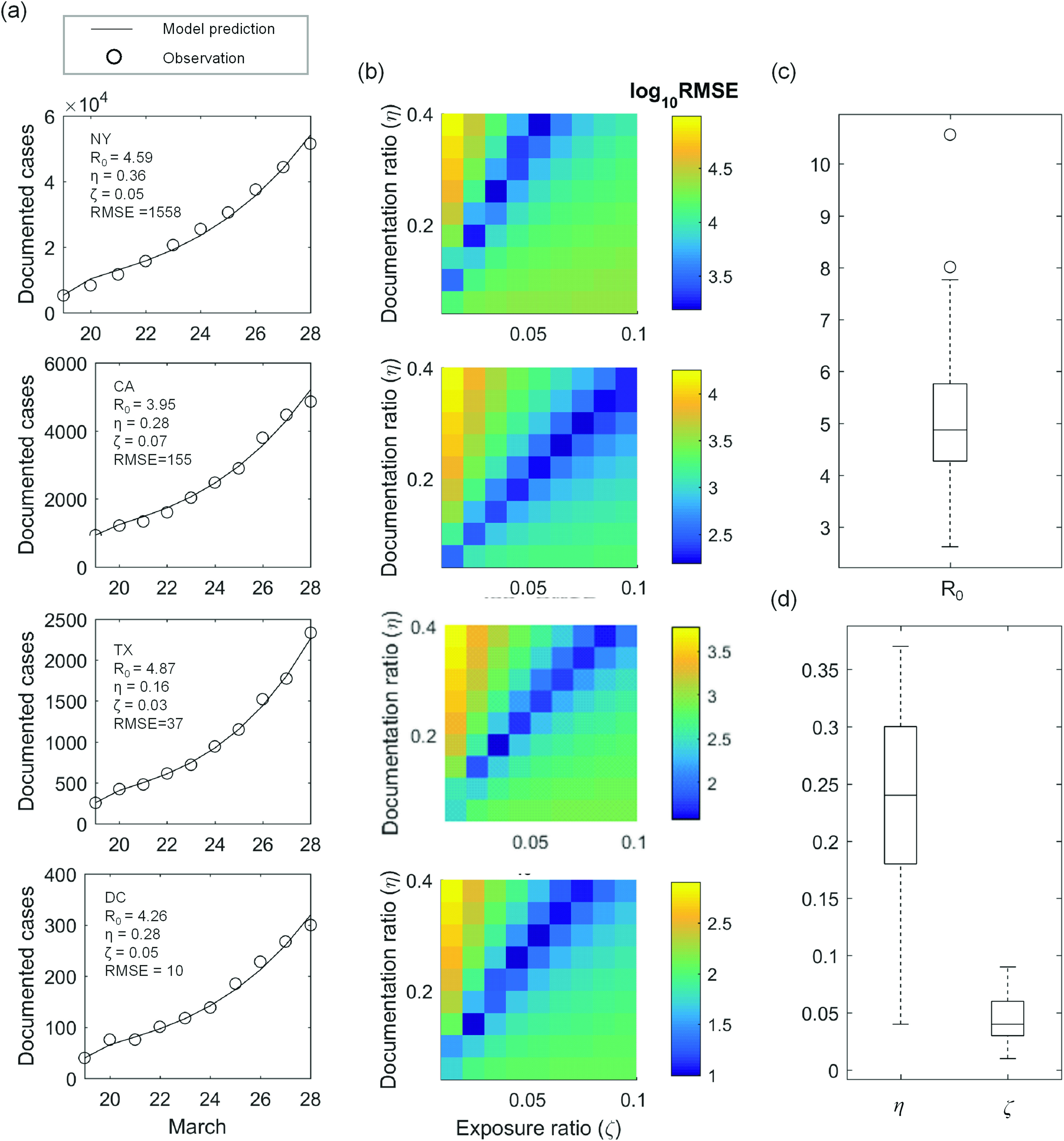
Model calibration between March 19 and 28. (a) Daily observation of documented active COVID-19 cases (circle) is compared with the best-fit model (line). The parameter combination leading to the best fit is outlined in each subpanel. (b) Root-mean-square-error (RMSE) between observation and model prediction is plotted as a function of documentation ratio (η) and initial exposure ratio (ζ) for each corresponding state. The basic reproduction ratio $\left(R_{0}\right)$ is held constant here, taking the values outlined in the respective subpanels in (a). (c) Distribution of $R_{0}$ across the 52 locations. (d) Distribution of ηandζ across the 52 locations. In (c) and (d), center of the box represents state-wise median values. Edges of the box represent the 25th and 75th percentile. Whiskers extend to the extreme data points not considered outliers, and outliers are represented by a circle symbol.

Figures 2(a) and 2(b) show the iterative inference results of COVID-19 epidemiological parameters. Figure 2(a) compares the $j_{n}^{\;r}(t)$ predicted by the best-fit model (line) with the observed epidemic trends in New York, California, Texas, and Washington DC (refer to supplementary material for the complete inference results for all 52 locations). Figure 2(b) shows the combination of $\eta_{n}\;\text{and}\;\zeta_{n}$ that gives rise to the minimum RMSE under a fixed $R_{0,n}$ [taking values outlined in the respective subpanels in (a)]. Figures 2(c) and 2(d) show the distribution of $R_{0,n},\;\eta_{n},\;\text{and}\;\zeta_{n}$ for all 52 locations. The nationwide median value of $R_{0}$ is found to be about 4.87 with the 25th and 75th percentile taking values of 4.27 and 5.76, respectively. These values are consistently larger than that reported in the other modeling studies conducted on the COVID-19 epidemics in China.^33^ This greater infectiousness could be due to the absence of public awareness and effective intervention in the US at the early stage of an epidemic outbreak. The nationwide median values of ηandζ are found to be about 0.24 and 0.04, respectively. The η≈0.24 is in good agreement with the WHO situation report No. 34^31^ which identifies that about 80% of all infected cases are undocumented.

### B. Indefinite-time social distancing

We estimate the demand on medical systems across the country by assuming that no effective containment intervention will take place and the epidemic will progress following the trend observed between March 19 and 28. In the context of this work, we interpret the burden on the medical resource using the local demand-to-supply ratio for hospital beds $\left(y_{H,n}\right)$ at an epidemic peak,

$$y_{H,n}=N_{n}\sum_{i}j_{n,i}^{*}P_{H,i}/X_{H,n}.$$ (2)

**FIG. 3.**
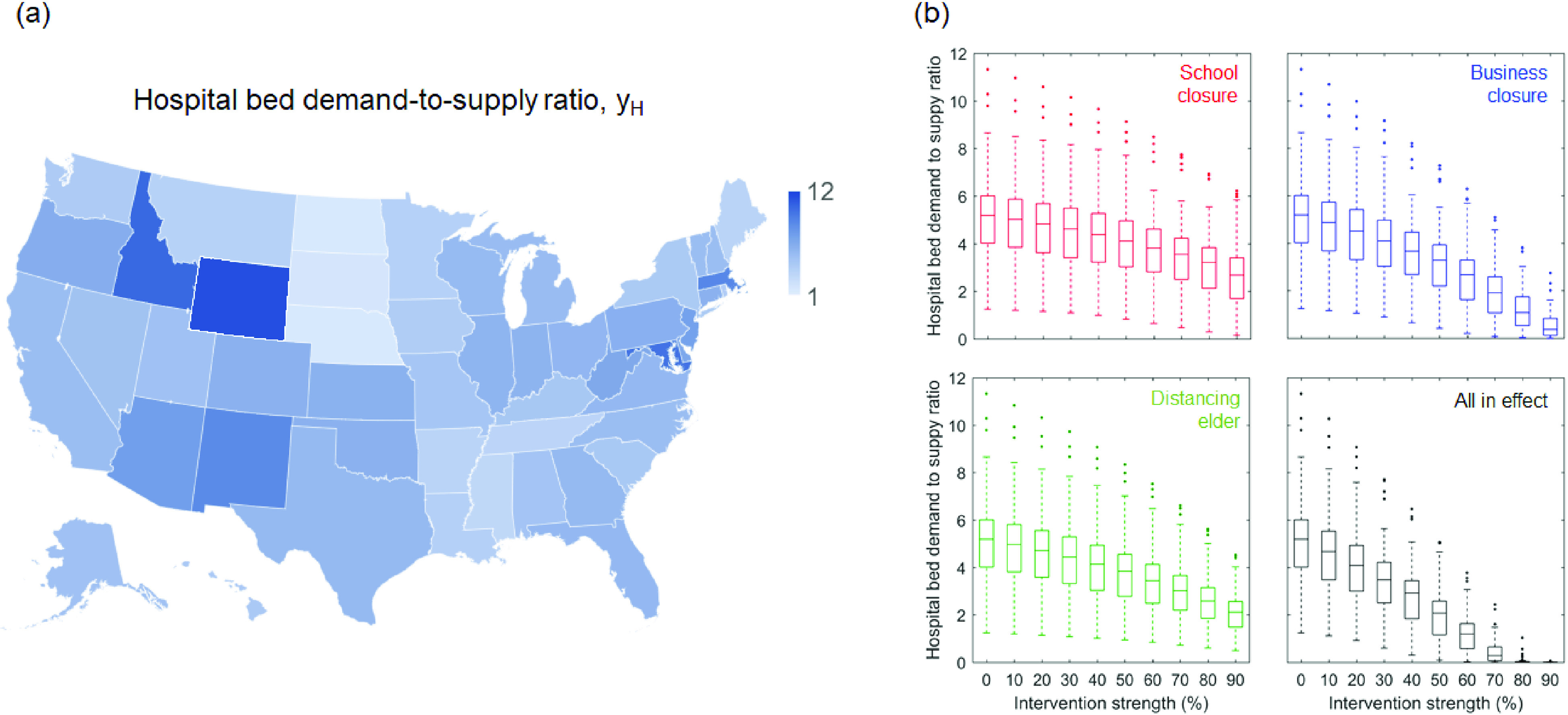
Effectiveness of various social distancing interventions in reducing hospital demand-to-supply. (a) Shows the estimated demand-to-supply ratio of hospital beds $\left(y_{H}\right)$ at state-wise epidemic peaks. (b) Demand-to-supply ratio of hospital bed $y_{H}$ is plotted as a function of intervention intensity (φ)—defined as the percent reduction in the exposure time of individuals targeted by the intervention. The center of the box represents state-wise median values of $y_{H}\;\text{and}\;y_{ICU}$. Edges of the box represent the 25th and 75th percentile. Whiskers extend to the extreme data points not considered outliers that are represented by dot symbols.

Here, $j_{n,i}^{*}$ is the peak value of $j_{n,i}(t);N_{n}$ is the total population of state n^30^; and $P_{H,i}$ denotes the age-specific rate at which COVID-19 patients require hospitalization (data tabulated in the supplementary material of Ref. 13); $X_{H,n}$ denotes the state-wise numbers of available hospital beds (data tabulated in the supplementary material of Ref. 34). One should note that the $y_{H,n}$ can be overestimated because the undocumented cases are constituted in part by asymptomatic and/or untested COVID-19 carriers, who might not require hospitalization. Figures 3(a) and 3(b) color each US state according to its corresponding $y_{H,n}$ value. If the epidemic progression remains unhalted, then the state-wise hospital beds could be overwhelmed by up to 12 times. Similar trends were also observed in the evolution of the demand-to-supply ratio of intensive care unit (ICU) beds, which can be found in the supplementary material of this work.

Figures 3(c) and 3(d) evaluate the effectiveness of various social distancing practices in balancing the medical demands and supply. Herein, the decreases in the peak $y_{H}$ are plotted as functions of the social distancing intensity (φ)—defined as the percent reduction in time-of-exposure $T_{i,k}$ of the targeted age-group members. For example, school closure reduces the values of $T_{i,k}$ elements that are associated with individuals aged 1–19 years; and business closure and distancing the elder reduce $T_{i,k}$ for those aged 20–59 years and those aged 60-year-and-above, respectively. Formally, this treatment is described with the following time-of-exposure matrix $\left(T_{i,k,\text{intv}}\right)$ modified per the type of social distancing practice,

$$\begin{array}{r} T_{i,k,\mathrm{int}v}=\varphi T_{i,k}\left\{ \begin{array}{l} \;for\text{\textbackslash{}; }i\leq 2\text{\textbackslash{}; }or\text{\textbackslash{}; }k\leq 2,\text{\textbackslash{}; school\textbackslash{}; closure}\\ for\text{\textbackslash{}; }i\in [3,\text{\textbackslash{}; }6]\text{\textbackslash{}; }or\text{\textbackslash{}; }k\in [3,\text{\textbackslash{}; }6],\text{\textbackslash{}; business\textbackslash{}; closure}\\ \;for\text{\textbackslash{}; }i\geq 7\text{\textbackslash{}; }or\text{\textbackslash{}; }k\geq 7,\text{\textbackslash{}; distancing\textbackslash{}; elder}\\ \;\;\;\;\;\forall i,\forall k,\text{\textbackslash{}; all\textbackslash{}; practices\textbackslash{}; in\textbackslash{}; effect} \end{array}\right.. \end{array}$$ (3)

The modification of the time-of-exposure matrix lasts for the duration of the epidemic. The side-by-side comparison in Fig. 3 shows that business closure is the most effective practice, possibly because it targets at the majority of the population. At a fixed φ level, elder distancing achieves a better outcome than school closure. However, none of these practices could curb COVID-19 adequately if they are implemented separately. Instead, a wholesale social distancing with all practices in effect must be taken, and it takes at least an intervention intensity φ=70% to reduce the medical demands to a balanced level under the premise that the social distancing can last for indefinitely long time.

### C. Finite-time social distancing

**FIG. 4.**
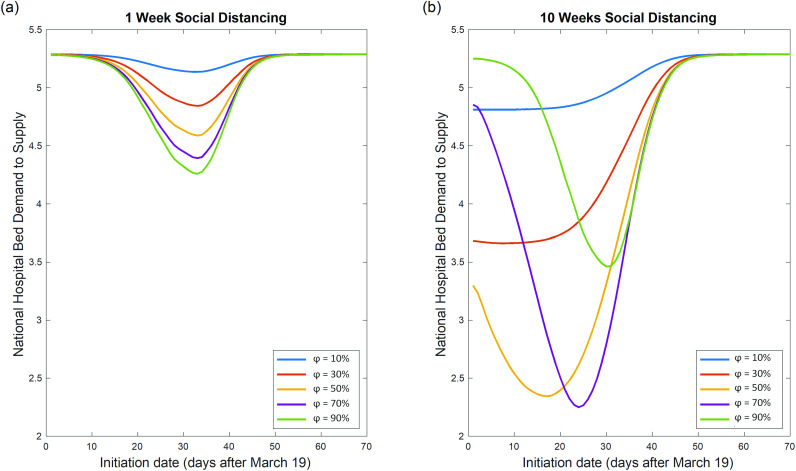
Interplay of intensity φ and implementation timing of finite-time social distancing. (a) Nationwide hospital bed demand-to-supply ratio $\left(\bar{y_{H}}\right)$ is plotted as a function of implementation timing for various social distancing intensities with a duration of 1 week. For social distancing lasting 1 week, stronger social distancing could lead to decreased medical demand if social distancing is implemented at the optimal time. (b) Nationwide hospital bed demand-to-supply ratio $\left(\bar{y_{H}}\right)$ is plotted as a function of implementation timing for various social distancing intensities for a duration of ten weeks. For social distancing lasting ten weeks, stronger social distancing does not necessarily lead to decreased medical demand, even if social distancing is implemented at the optimal time $\left(t_{I}\right)$.

A prolonged period of social distancing could have devastating socio-economic implications that outweighs its benefits.^35,36^ If society can only afford a finite-time social distancing, it is of utmost importance to understand *when* and for *how long* the intervention should be put into effect, so as to maximize the net benefit. Figures 4(a) and 4(b) plot the effectiveness of finite-time social distancing as functions of intervention duration and implementation timing for various φ. (Here, the nationwide demand-to-supply ratio of medical resources: $\bar{y_{H}}=\sum_{n}y_{H,n}X_{H,n}/\sum_{n}X_{H,n}$ are used to benchmark intervention effectiveness.) The trends in Figs. 4(a) and 4(b) show that unless the intervention could last indefinitely, a premature implementation could be counterproductive. It is also evident that the duration of social distancing greatly affects the optimal value of φ. Figure 4(a) shows that for social distancing lasting for one week, increased φ leads to decreased medical demands. However, Fig. 4(b) shows that for social distancing lasting for ten weeks, there is an optimal value of φ ≈ 70% which corresponds to the maximum achievable reduction in medical demands. Increasing φ beyond this point decreases the effectiveness of the intervention. The minimum “valley” points corresponding to each curve denote the optimal $t_{I}$ as well as the corresponding maximum achievable reduction in $\bar{y_{H}}$.

**FIG. 5.**
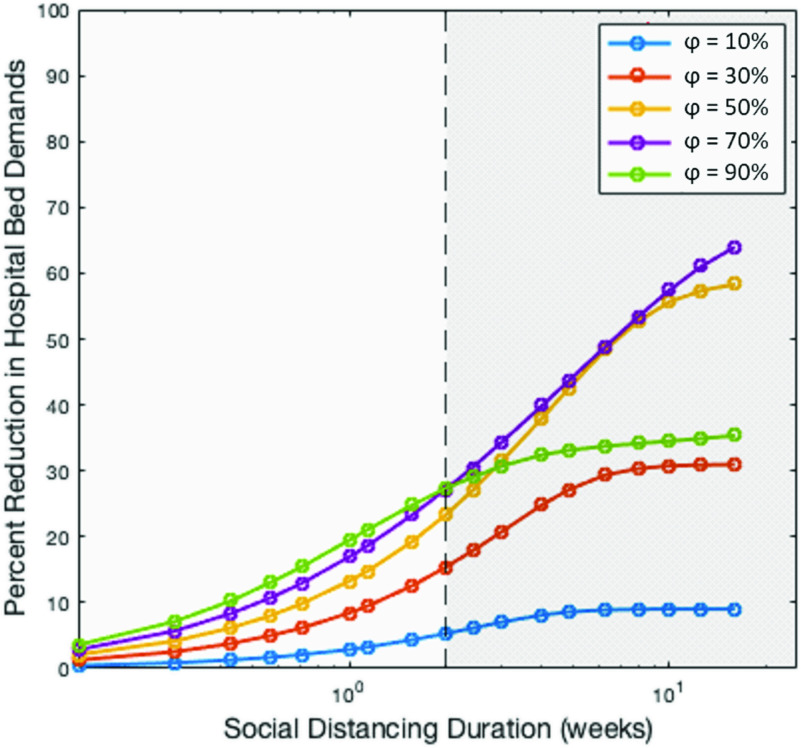
Exponentially diminishing benefits of social distancing. Semi-log plot for the relationship between social distancing duration and the corresponding maximum attainable decrease in medical demands for social distancing of various intensities. The emergence of two regimes becomes clear, separated into short-term (duration <2 weeks) and long-term (duration ≥ 2 weeks, gray shaded region). For short-term social distancing, stronger social distancing (denoted by increasing intensity φ) leads to a monotonous decrease in medical demands. For long-term social distancing, the largest reduction in medical demands is achieved when φ ≈70%, beyond which the benefits of social distancing diminish.

In Fig. 5, we highlight the diminishing marginal benefits of social distancing by plotting the maximum achievable reduction in $\bar{y_{H}}$ as a function of social distancing duration (weeks). The figure clearly exhibits an emergence of two regimes. The first regime corresponds to social distancing duration <2 weeks (hereafter short-term social distancing). Within this regime, increased φ leads to a proportionate reduction in medical demands. The second regime corresponds to social distancing with duration ≥2 weeks (hereafter long-term social distancing). Within this regime, a linear decrease in medical demands is achieved at the cost of an exponentially increasing social distancing duration. Increasing φ >70% yields a significantly lower than expected reduction in medical demands. This decreased effectiveness of increasing intensity and duration of social distancing could be best described by the law of diminishing returns, where the benefits of the intervention dwindle with more units invested. It should be noted that if the social distancing duration were to be further increased, then the results would converge to those discussed in Sec. III B.

**FIG. 6.**
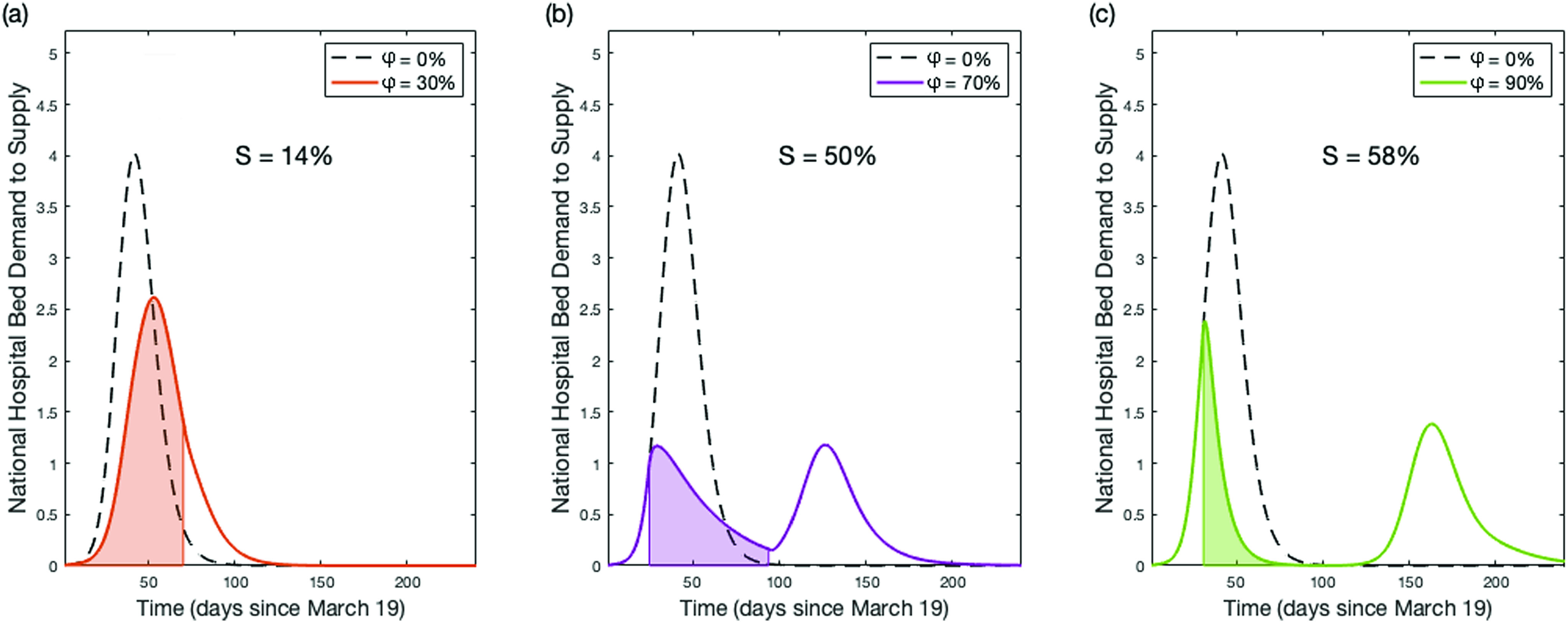
Epidemic dynamics of ten-week social distancing with varying intensities φ. Epidemic dynamics for ten weeks social distancing initiated at $t_{I}$ with φ = 30% (a), φ = 70% (b), and φ = 90% (c). Shaded regions denote the period where social distancing is in effect. The magnitude of the second peaks seen in panels (b) and (c) are controlled by the percentage of susceptible individuals (S) within the population when social distancing ends. If the susceptible population is very large when social distancing ends, a second wave of infections will spread throughout the population.

We attempt to provide an explanation behind the phenomenon of reduced medical benefits for φ >70% observed during the long-term social distancing period. In Fig. 6, we explain the origin of this occurrence by elucidating the epidemic curves that evolve under a long-term social distancing period of 10 weeks for various φ. When φ ≥ 70%, there exists a large buildup of susceptible individuals, who remain prone to the infection at the end of the social distancing period. The possibility of this residual susceptible population to emerge as infectious with time remains very high. Thus, one cannot rule out the possibility of a second wave of infections in the USA after the initial wave of pandemic dies out.^12^ This non-linear dynamics of intensity and duration indicates that if social distancing cannot be maintained indefinitely, its intensity, implementation time, and duration must be considered carefully in order to maximize its benefits. Another implication of our findings is that a premature removal of strict social distancing measures could lead to a large second wave of infections. The non-linear behavior of finite-time social distancing warrants the aggressive co-implementation of other interventions such as contact tracing. Before re-opening of normal social-economic activities, efforts need to be taken on identifying the potential COVID-19 carriers and physically isolating them from the large susceptible population, which will ultimately prevent the onset of second wave of infection.^37^

### D. Intermittent social distancing

**FIG. 7.**
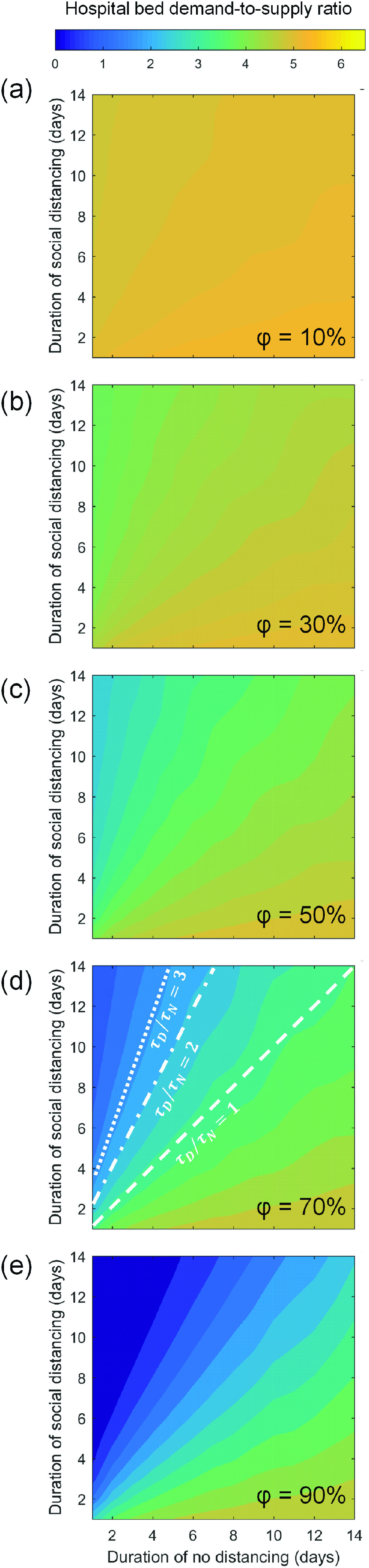
Effectiveness of intermittent social distancing. Nationwide hospital bed demand-to-supply ratio $\left(\bar{y_{H}}\right)$ is plotted as a function of social distancing phase duration $\left(\tau_{D}\right)$ and normalcy phase duration $\left(\tau_{N}\right)$. Each subpanel (a)–(e) shows the $\bar{y_{H}}\;$distribution under a fixed intervention intensity φ. In panel (d), the intermittent arrangements with $\tau_{D}/\tau_{N}$** = **1, 2, and 3 are marked with the dashed line, the dashed-dotted line, and the dotted line, respectively, so as to highlight the determining role of the intermittent ratio $\tau_{D}/\tau_{N}$ under a fixed φ.

We next evaluate the effectiveness of intermittent social distancing strategy—an arrangement comprising of alternating phases of social distancing and no-distancing that last for variable durations—as a sustainable solution.^38^ Figure 7 plots the hospital bed demand-to-supply ratio as a function of social distancing phase duration $\tau_{D}$, no-distancing (or normalcy) phase duration $\tau_{N}$, and intervention intensity φ. A comparison among each subpanel of Fig. 7 indicates that the effectiveness of intermittent social distancing is positively correlated with φ. Furthermore, when φ is fixed, the effectiveness of intermittent social distancing is determined by $\tau_{D}/\tau_{N}$—the characteristic intermittent distancing-to-no-distancing ratio. For instance, in Fig. 7(d), the intermittent arrangements corresponding to $\tau_{D}/\tau_{N}$ = 1, 2, and 3 are marked with lines of various styles. One could observe that the arrangements with the same $\tau_{D}/\tau_{N}$ result in the same demand-to-supply ratio outcome.

**FIG. 8.**
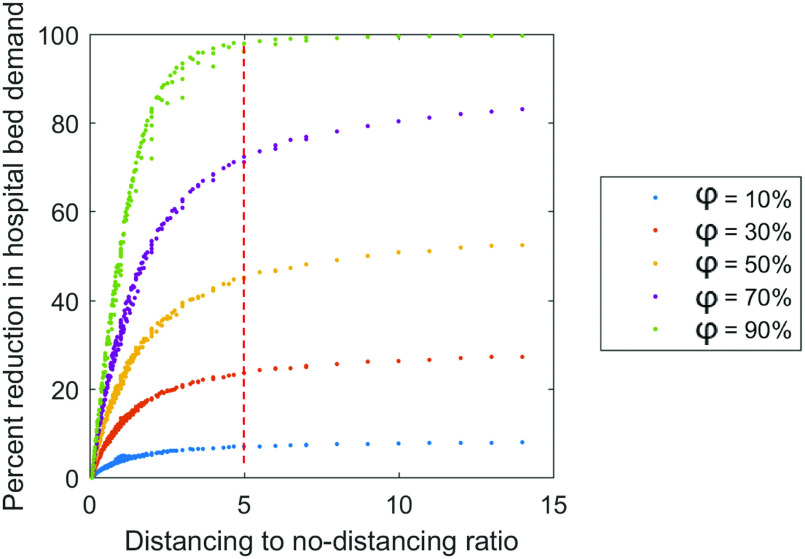
Intermittent distancing to no-distancing duration ratio $\left(\tau_{D}/\tau_{N}\right)$ and reduction in medical demands. At any intervention intensity φ, for 1 ≤ $\tau_{D}/\tau_{N}$ ≤ 5, the effectiveness of social distancing—measured by the percent reduction in hospital bed demand—increases rapidly with an increasing $\tau_{D}/\tau_{N}$. The effectiveness wanes off around $\tau_{D}/\tau_{N}\approx 5$.

The determining role of the characteristic $\tau_{D}/\tau_{N}$ ratio is further elucidated in Fig. 8, wherein the percent reduction in hospital bed demand is plotted as a function of $\tau_{D}/\tau_{N}\;\text{and}\;\varphi$. These observed universal trends again imply that the marginal benefits of social distancing would diminish over a prolonged time. For 1 ≤ $\tau_{D}/\tau_{N}$ ≤ 5, the effectiveness of social distancing—measured by the percent reduction in medical demands—increases rapidly with an increasing $\tau_{D}/\tau_{N}$. Beyond the critical point of $\tau_{D}/\tau_{N}\approx 5$, the increase in effectiveness due to a further increase in $\tau_{D}/\tau_{N}$ becomes negligible. Such an observation holds valid regardless of φ. Take intermittent social distancing with φ = 70% as an example: an effective intermittent social distancing strategy would allow people to behave as normal for one day, followed by five days of social distancing, and then repeat. This strategy could alleviate medical burden up to 70% and could avoid some of the burdens of long-term social distancing. This behavior is also well described by the law of diminishing returns; there is an optimal distancing-to-no-distancing ratio, beyond which the time invested becomes greater than the benefits gained.

## IV. CONCLUSION

In this work, we provide a comprehensive systematic analysis of the effectiveness of three social distancing methods in alleviating the burden of the COVID-19 pandemic on nationwide medical resources. Our baseline scenario represents a continuation of the epidemic dynamics observed between March 19 and 28, during which a rapid surge of COVID-19 cases is observed in the United States and the effect of social distancing is yet to manifest. Our findings suggest that under such a baseline condition, the state-wise hospital could be overwhelmed by up to 12 times. Indefinite-time social distancing is found to balance the medical demand-to-supply at epidemic peak provided a 70% reduction in the time-of-exposure of the population within all age groups is achieved. Finite-time social distancing is found to follow two regimes; short-term (duration <2 weeks) and long-term (duration ≥2 weeks). Short-term social distancing is characterized by a proportionate reduction in medical demands for increased social distancing intensity (φ). Long-term social distancing is characterized by an optimal φ (∼70%), which yields the largest reduction in medical demands. Long-term social distancing is also characterized by a linear decrease in medical demands for exponentially increasing social distancing duration. The non-linear dynamics between φ and hospitalization demand emphasize that precautions need to be taken when lifting strict social distancing measures abruptly, so as to prevent the onset of a second wave of infection. Secondary interventions such as contact tracing are necessary for identifying and isolating the COVID-19 carriers from a potential large susceptible pool of the population. Intermittent social distancing is found to rapidly reduce medical demands, as long as the characteristic distancing-to-no-distancing ratio $\left(\tau_{D}/\tau_{N}\right)$ does not exceed 5. If $\tau_{D}/\tau_{N}$ is increased beyond 5, the benefits begin to wane significantly. Finite-time social distancing and intermittent social distancing are both found to be effective social distancing strategies in mitigating medical demands related to the COVID-19 pandemic. However, the implementation of these strategies must be carefully considered, as their benefits are found to be well described by the law of diminishing returns. Findings from this study may also apply to other regions of Europe, as well as Asia, where social distancing measures have been in effect to slow the epidemic spread.

## SUPPLEMENTARY MATERIAL

See the supplementary material for the following datasets:
•Time-of-exposure matrix: The daily average time-of-exposure of the age-stratified American population. Units: minutes per day.•Interstate mobility matrix: Monthly resolved datasets quantifying the interstate mobility pattern in the USA. The matrix elements represent the probability that a passenger leaving a departure state (row) ends up in a destination state (column). Units: dimensionless.•Model calibration results: The state-wise epidemiological parameters that lead to the best fit between model prediction and observed epidemic trend. Units: Basic reproduction ratio, documentation ratio, exposed ratio: dimensionless; RMSE: number of people.•Hospitalization and critical condition rate: The age-specific probability by which an infected individual requires hospitalization, and the age-specific probability by which a hospitalized COVID-19 patient develops into critical condition. Units: dimensionless.•State-wise population: State-wise population and demographic composition. Units: Population: number of people; fraction of population within each age group: dimensionless.•State-wise medical resources: State-wise number of available hospital beds and ICU beds. Units: number of beds.

## AUTHORS’ CONTRIBUTIONS

P. L. and P. B. contributed equally to this manuscript.

## Data Availability

The data that support the findings of this study are available in the supplementary material and from the Johns Hopkins University COVID-19 Tracking website, Ref. 8.
